# The Application and Outcome of Standard of Care Treatment in Elderly Women with Ovarian Cancer: A Literature Review over the Last 10 Years

**DOI:** 10.3389/fonc.2016.00063

**Published:** 2016-03-24

**Authors:** Steven J. Gibson, Gini F. Fleming, Sarah M. Temkin, Dana M. Chase

**Affiliations:** ^1^The Division of Gynecologic Oncology, University of Arizona Cancer Center, St. Joseph’s Hospital and Medical Center, Creighton University School of Medicine, Phoenix, AZ, USA; ^2^Department of Medicine, The Division of Hematology/Oncology, Knapp Center for Biomedical Discovery, The University of Chicago, Chicago, IL, USA; ^3^The Department of Gynecology and Obstetrics, The Johns Hopkins Hospital, Johns Hopkins University, Baltimore, MD, USA

**Keywords:** ovarian cancer, elderly, age, treatment, care, chemotherapy, outcomes

## Abstract

The rising number and increasing longevity of the elderly population calls for improvements and potentially a more personalized approach to the treatment of cancer in this group. Elderly patients frequently present with a number of comorbidities, complicating surgery and chemotherapy tolerability. In the case of ovarian cancer, elderly women present with more advanced disease, making the issue of providing adequate treatment without significant morbidity critical. Most studies support the application of standard of care treatment to elderly women with ovarian cancer, yet it seems to be offered less frequently in the elderly. The objective of this review is to examine the application and outcome of standard of care treatment in elderly women with ovarian cancer. The aim is to ultimately improve the approach to treatment in this group.

## Introduction

The elderly population, defined as 65 years and older, is expected to reach 80 million in the United States over the next two decades (1). Ovarian cancer is common among older women, with estimates suggesting that half of the women living with ovarian cancer are over 65 years old (2, 3). Over two-thirds of new cases are in women over 55 years old, with the median age at diagnosis being 63 (4). While some cancers, such as breast, generally become more indolent with increasing age, the reverse is seen in ovarian cancer (5), resulting in increasing complexity of treatment. Many studies continue to investigate why survival in the elderly differs so much from that of younger cancer patients. Freyer and colleagues in a 2013 review proposed various theories to explain these higher death rates. They proposed that this could be due to more aggressive cancer with advanced age, inherent resistance to chemotherapy, multiple concurrent medical problems, and physician and healthcare biases toward the elderly that lead to inadequate surgery, less than optimal chemotherapy, and poor enrollment in clinical trials (6).

**Figure 1:**
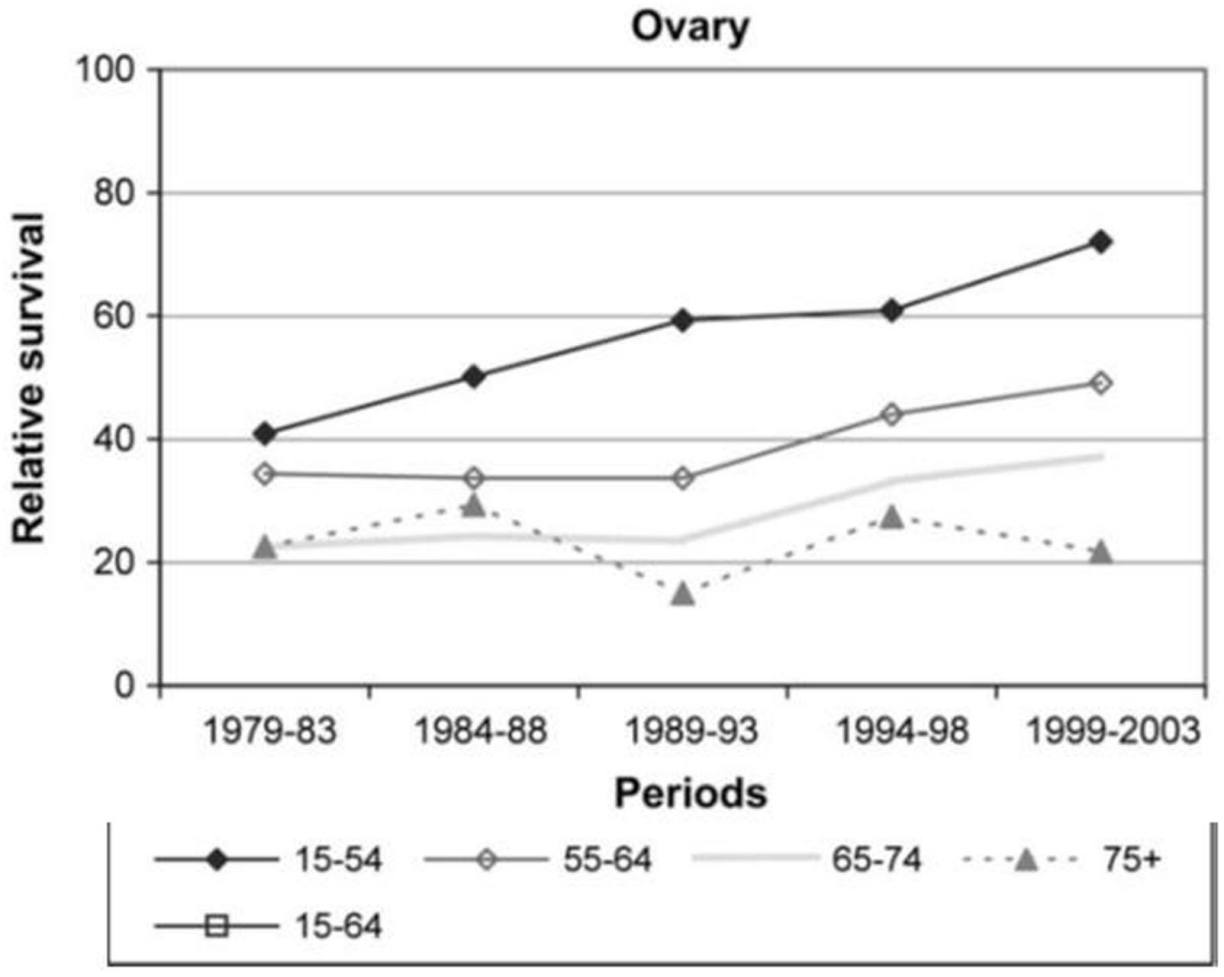
**Relative survival rates by age in ovarian cancer patients over 20-year study period (8)**.

Both treatment administered and outcomes observed in the elderly ovarian cancer population have differed from their younger counterparts. For example, a Surveillance, Epidemiology and End Results (SEER) data analysis of almost 10,000 elderly women (>65 years) between 1991 and 2007 found that over the past couple of decades, primary surgery had significantly decreased from 63.2 to 49.5%, while primary chemotherapy doubled from 19.7 to 31.8% (7), as later described in Figure 3. In addition, a German study found that ovarian cancer patients aged 15–54 had a strong continuous trend of improving survival, as did patients aged 55–74, yet elderly patients >75 years saw no improvement in survival during the 1979–2003 study period. As the age gradient substantially widened over time, reaching a relative survival difference of 50% between the two groups, it was the strongest age gradient observed among 15 examined cancers after a 20-year analysis Figure 1 (8). Both of these examples illustrate how treatment and outcomes continue to differ from women in younger age groups. Acknowledging these differences as well as the deficits in the literature is the objective of this review. Once these deficits are better defined, research can be initiated.

## Methods

A PubMed literature review was conducted using various combinations of the following search terms: “ovarian cancer,” “elderly,” “gynecologic(al) cancer(s),” “treatment,” and “care.” The articles were screened for original articles and reviews published between 2005 and 2015. Only English articles were reviewed. Seventy-six articles met these inclusion criteria.

### Baseline Variables Predictive of Outcome

Several studies have examined the diagnosis of ovarian cancer in elderly patients. Ovarian cancer is a disease of the elderly as the average age of diagnosis is 63. For example, in a large study by Poynter et al., older age at baseline was the only significantly associated risk factor for developing ovarian cancer in elderly women (9). Although ovarian cancer is typically diagnosed at an advanced stage, an even larger proportion (80%) of elderly women present with Stages III–IV disease (6). Analysis of SEER data from 1988 to 2001 found that in over 28,000 women, younger women were two to three times more likely to be diagnosed with early-stage (I–II) disease than their elderly counterparts (10). Other studies have shown similar results (11). A separate SEER analysis of 4,000 advanced ovarian cancer patients diagnosed between 1992 and 1999 illustrates the consequence of elderly women being diagnosed with higher stages of disease, as survival is significantly associated with stage (12). These SEER data are also described in Figure 3. When comparing the elderly (65–74 years) with the very elderly (≥75 years), increased age was also associated with advanced stage and higher grade (13). While other studies may not support this trend (10, 11, 14–18), the majority suggest the importance of age as a baseline that affects treatment outcomes.

Comorbidities are common in all elderly women, regardless of cancer status, but women with ovarian cancer in general had a much higher incidence of comorbidities than cancer-free women. Because of this, understanding the role of comorbid conditions in elderly ovarian cancer treatment and outcomes will be crucial for optimal personalized treatment in this group (19). The complexity of ovarian cancer treatment, including surgery and chemotherapy, may limit the ability of elderly women with comorbidities to tolerate radical surgery and toxic therapeutic regimens.

Because of this, having a prognostic tool to predict the impact of covariates on overall survival (OS) would be of value in this complex patient population. The GINECO study used three separate phase II trials to develop a new prognostic tool, called the geriatric vulnerability score (GVS), which can be utilized to predict survival in elderly (≥70 years) patients with advanced ovarian cancer. The best-fitting model delivered a survival score equal to exp(0.327 × GVS), where the GVS is the sum of the following of a scale of 0–5 (each with a value of one): albumin <35 g/l; activities of daily living (ADL) score <6; instrumental activities of daily living (IADL) score <25; lymphopenia <1 G/l; and Hospital Anxiety and Depression Scale (HADS) >14. The GVS was significantly differentiated between two groups: those with a score <3 having an 82.1% chemotherapy completion rate, while those over 3 only observed 65.5% completion rates. Women with a GVS ≥3 were over twice as likely to have grade ≥3 non-hematological toxicities, twice as likely to have serious adverse event, and experienced more unplanned hospital admissions (20).

### Primary Surgical Treatment

Initial therapy for ovarian cancer following diagnosis includes a combination of surgery and chemotherapy. Patients with the best prognosis include those who undergo surgical cytoreduction to no gross disease and receive platinum and taxane-based chemotherapy, with some receiving treatment through an intraperitoneal infusion.

**Figure 2:**
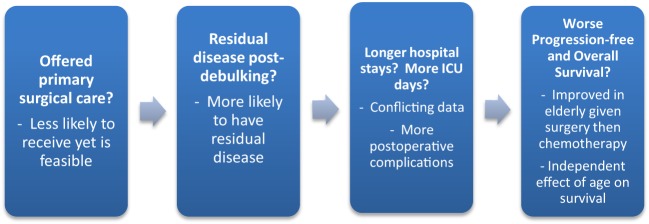
**Primary surgical treatment for elderly women with ovarian cancer**.

**Figure 3:**
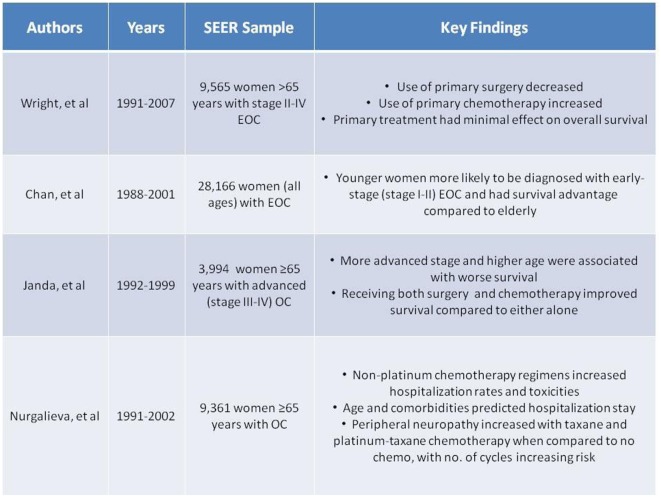
**Summary of Selected Surveillance, Epidemiology and End Results (SEER) data (7, 10, 12, 54, 55)**.

Many recent studies have examined how primary treatment in the elderly compares to younger women with ovarian cancer (Figure 2). For example, in an analysis of over 10,000 patients with ovarian cancer, the elderly were less likely to receive comprehensive surgical care, as defined by International Classification of Disease, 9th Revision (ICD-9) diagnosis and procedure codes (21). Similarly, an analysis of over 23,000 advanced ovarian cancer patients in the Netherlands found that about one-third of elderly patients received no therapy (22). Other studies support the trend in elderly women receiving suboptimal treatment (11, 13, 15, 18, 22–27). A 961-patient study even found elderly age to be independently predictive of not receiving cytoreductive surgery and standard combination chemotherapy (24). A SEER analysis of 28,165 women with ovarian cancer found that younger women were significantly more likely to undergo primary surgical procedures than the elderly (10) (Figure 3). This was supported by other studies as well (23). Although some studies did not confirm this difference in treatment based on age (16, 17, 28, 29), the bulk of the data demonstrates a disproportionate number of elderly women receiving suboptimal treatment.

Interestingly, most studies do indicate that optimal treatment in the elderly is feasible and acceptable, with similar outcomes observed between age groups (15, 16, 23, 24, 27, 30–32). After adjusting for age and stage of ovarian cancer, optimal treatment had a significant impact on survival, suggesting that focus should be placed on optimal treatment for patients of all ages with ovarian cancer (32). When comparing the elderly and very elderly, multiple studies found no significant difference in perioperative complications, with progression-free survival (PFS) and OS being similar (16, 29, 33).

While surgery may be feasible, the residual disease volume post-debulking surgery has been found to be higher for elderly patients, which significantly impacts PFS and OS (11, 16, 27, 29, 34–36). A Mayo Clinic study of women with advanced ovarian cancer found that residual disease had a larger and more significant impact in the very elderly, with a fourfold decrease in median survival when compared to younger patients (37). With perhaps a higher rate of residual disease left at the time of surgery and a greater impact on outcomes, such as survival, some question the use of this aggressive treatment in this population.(23, 38), However, elderly women who do undergo primary debulking surgery have better disease-free survival and OS than those who had interval debulking (33). Yet, the elderly have also been found to have a statistically higher rate of large bowel resection than their younger counterparts (15). Hospitalization data are conflicting, with some studies showing days of hospitalization or ICU stay to be longer in the elderly (28, 33), while others contradict this (23). Discrepancies are likely influenced by selection bias. Further investigation with larger sample sizes is warranted.

In a Maryland state-wide study, it was observed that university-type hospitals were significantly less likely to have admitted the elderly patients when compared to younger patients, with the majority of elderly admissions being for surgeries under emergency conditions. Interestingly, older women with ovarian cancer were also significantly more likely to have a different operating surgeon than the attending physician of record. The elderly also had a higher adjusted cost of hospital-related care with more billable procedures, and a 30-day mortality rate 2.3 times higher than that of younger patients. When analyzing surgeon-type, elderly patients of high-volume surgeons (≥10 cases/year) billed twice as manyprocedures, had nearly a tripled cost of hospital care, and twice as many comorbidities as younger patients. Interestingly, 62% of elderly women saw high-volume surgeons even though these surgeons only represented 3.4% of the surgeons in the study. Similarly, while only 18.4% of hospitals in the study were considered high-volume (≥20 cases/year), the majority (60%) of elderly women were treated at these hospitals and had more procedures billed and more comorbidities (28).

Earle et al. examined the impact of surgeon specialty on outcome for 3,067 elderly ovarian cancer patients and found that those treated by gynecologic oncologists had superior outcomes to those treated by general gynecologists or general surgeons. Advanced-stage disease patients were more likely to undergo debulking if the surgery was performed by a gynecologic oncologist as opposed to a general gynecologist or general surgeon. Survival among patients operated on by gynecologic oncologists or general gynecologists was far better than that among patients operated on by general surgeons (39). This is supported by another study, which found surgeries performed by non-gynecologic oncologists observed the risk for mortality to double (31).

A study of 2,087 women with ovarian cancer from the American College of Surgeons National Surgical Quality Improvement Program (ACS-NSQIP) database found there to be a high risk of perioperative mortality and morbidity within 30 days in elderly patients with ovarian cancer (40), as supported by other data outcomes on high risk women (12, 38). The elderly were also more likely to develop pulmonary and septic complications, and were nine times more likely to die and 70% more likely to develop complications within 30 days of surgery (40). Similarly, Moore et al. demonstrated elderly patients may not tolerate surgery and combination chemotherapy, paying a high price in post-operative complications and death (41).

Different types of procedures have been examined in the elderly as well. One study examined the effects of interval debulking after neoadjuvant chemotherapy and hyperthermic intraperitoneal chemotherapy (HIPEC) in the elderly with advanced ovarian cancer as an alternative to initial complex surgery. This study found that the elderly group did not receive benefit from the interval cytoreduction with HIPEC treatment and instead experienced postoperative morbidity, with the most common being grade 4 hemoperitoneum and grade 3 intra-abdominal fluid collection in 22.2% of women (17). Another study that evaluated the feasibility and safety of extensive upper abdominal surgery (EUAS) in elderly patients with advanced ovarian cancer found no significant difference to that of younger patients, and concluded that EUAS procedures are feasible in this elderly population (35).

The impact of nutritional status on survival outcomes was examined by Alphs et al., who found that poor nutrition was associated with poor survival outcomes. Albumin levels ≥3.7 g/dl were associated with a 40% reduction in risk of mortality in the elderly population and, overall, elderly women had a 2.6-fold greater risk of mortality when compared with younger women (31).

Most conflicting, however, was univariate and multivariate analysis on the impact of age on treatment outcomes. Multiple studies showed increased age was independently associated with a significant, negative impact on survival (10, 12, 18, 22, 25, 31, 34, 40, 42, 43), while others show no significant, age-related impact on survival (15, 16, 23, 24, 26, 27, 30–32). Some of these studies include disease-free survival outcomes, which may help explain the conflicting data. The Centers for Disease Control and Prevention (CDC)-funded cancer registries examined the impact of age on survival in 2,367 women with ovarian cancer. Survival rates were lower in the oldest groups, especially in those with advanced disease. For example, 3-year survival in patients with stage IV was only 13% in the elderly compared to 50% in women under 35 years old. The adjusted risk of death doubled from 40% in younger women to 80% in the elderly. The CDC confirmed the independent adverse effect of age on survival in this patient population (25).

Significant survival advantages were seen in the younger patients with early-stage disease, as young age was an independent prognostic factor for increased survival. Advanced-stage disease had poorer survival in the elderly (10). As defined above, optimal treatment includes cytoreductive surgery with combination chemotherapy, and a 1992–1999 SEER analysis of almost 4,000 advanced ovarian cancer patients supports this treatment with the observation that elderly patients who received both surgery and chemotherapy showed significantly improved survival compared to either treatment alone (12) (Figure 3). However, it is worth noting that these findings may be influenced by selection bias. A retrospective analysis also evaluated each treatment individually and found that primary surgery was more beneficial than primary chemotherapy on survival outcomes (30, 44).

While NACT in elderly women only demonstrated a trend to improved PFS and no improvement in OS, NACT benefits were clearly demonstrated in a 62-patient study. Nearly a threefold increase in the rate of cytoreduction to no macroscopic disease was seen in women who received NACT when compared to those without. The NACT patients also had significantly less blood loss during surgery and required fewer small bowel resections (45).

In a study of almost 600 women with ovarian cancer, elderly women had a much poorer prognosis, possibly related to the significantly higher incidence of suboptimal treatment in this group. While no significant difference in PFS was observed between the two groups, median OS was over twice as long in the younger population (18). With no difference in PFS observed, the difference in OS may instead be attributed to comorbidities preventing second- or third-line chemotherapy treatment as opposed to strictly the result of suboptimal treatment.

An analysis of the OVCAD consortium, including 275 patients with ovarian cancer, found that the postoperative 60-day mortality rate was 5.25-fold higher in the elderly than in younger patients. The elderly also had a significantly worse median PFS and OS. Interestingly, age itself was not a prognostic factor for PFS in multivariate analysis, reiterating the significant role of optimal treatment on survival outcomes in the elderly (26). These results also demonstrate the confounding impact of age, grade, and stage on PFS outcomes.

### Adjuvant Chemotherapy

The indications for using of standard adjuvant chemotherapy in the elderly are inconsistent. As noted above, whether age is an independent prognostic factor for survival is unclear. Some studies show increasing age to be significantly associated with poorer survival outcomes (37, 43, 46–48), while others demonstrate no significant differences in survival outcome among the elderly (11, 16, 49–51). Not receiving adjuvant chemotherapy was found to negatively impact OS (34), and having more than three chemotherapy cycles was found to be an independent prognostic factor for OS in the elderly (18). When comparing the elderly (70–75 years old) to the very elderly (>75 years old), there was no difference in toxicity, dose reduction, and treatment delay or discontinuation (16). Even given these data, suboptimal chemotherapy administration in the elderly continues to be observed in most studies (13, 24, 27, 50–53). However, the impact of selection bias on these data cannot be underestimated.

While it is apparent the elderly do not receive equivalent standard of care chemotherapy treatment as their younger peers, some studies suggest that the elderly do not tolerate this regimen (41, 43). In one study of 109 patients, elderly women were less likely to complete all planned cycles of intraperitoneal chemotherapy when compared to a younger cohort. In addition, more intravenous chemotherapy was completed by elderly women who were optimally debulked as compared to those with residual disease (49). Another study found that the very elderly were prescribed combination chemotherapy much less frequently than younger patients, had significant differences in delayed initiation of chemotherapy, and six-cycle completion rate was only half that of the younger group (47, 52). The very elderly also had a 30-day mortality rate fourfold that of their elderly counterparts (46).

Common chemotherapy toxicities in the elderly across multiple studies included: grade 3–4 hematologic and gastrointestinal toxicities (16) and grade 3–4 neutropenia (51), with the use of paclitaxel as an independent prognostic factor for worse survival and increasing toxicities (48). While these trends in toxicity among the elderly are worth noting, the small study sizes may be misleading, as many studies show no significant difference in toxicities between age groups (11, 14, 50, 52).

A SEER analysis from 1991 to 2002 found that non-platinum chemotherapeutic regimens (administered in 18% of women) had higher rates of hospitalizations for gastrointestinal and hematologic conditions or infections compared to platinum-based or platinum–taxane combination regimens in 9,361 elderly women with ovarian cancer. While age was a significant predictor for hospitalization due to infection and cardiovascular diseases, older age did not predict gastrointestinal and hematologic toxicities (54). A separate, larger SEER analysis among over 9,000 women with ovarian cancer during the same 1991–2002 period found taxane therapy to double, and platinum–taxane therapy to triple, the risk of peripheral neuropathy when compared to elderly not receiving chemotherapy treatment. Risk was greater with an increasing number of cycles. Monitoring of peripheral neuropathy in this patient population receiving these chemotherapy regimens is warranted (55). The results of both SEER analyses are summarized in Figure 3.

A National Cancer Institute Common Toxicity Criteria (NCI CTC) analysis found that younger women received standard-dose chemotherapy nearly three times as often as the elderly (52). One study examined dose-delay in chemotherapy among elderly ovarian cancer patients and found that it was associated with a decrease in OS, even after controlling for age, stage, residual disease, and number of chemotherapy cycles received. This is of significance, as elderly patients frequently require chemotherapy dose reductions and delays in administration, and multivariate analysis suggested that dose-delays are an independent factor associated with decreased OS (56). However, a retrospective, multi-center analysis demonstrated no difference in survival outcomes between the reduced-dose and standard-dose elderly patients, and with the elderly more commonly on reduced-dose regimens, the authors suggested that carboplatin/paclitaxel may be better tolerated and equally as effective in this elderly population (51).

The 779-patient AGO OVAR-3 phase III study evaluated first-line platinum/paclitaxel in ovarian cancer patients, and found that ECOG performance status 2, measurable disease, and early discontinuation of therapy were much more common in the elderly (14). Another analysis of the same study found that young patients achieved no residual tumor after surgery more often and had significantly better survival when compared to the elderly, even when comparing those that were completely debulked across ages (43).

In a study of over 450 women with ovarian cancer, elderly women were more likely to receive carboplatin monotherapy, while younger patients were more likely to receive paclitaxel-containing chemotherapy. Only about half of the elderly patients received 100% paclitaxel relative dose intensity (RDI), while over two-thirds of the younger patients did. While the median OS of younger patients was significantly longer than that of older patients, PFS did not differ significantly between the two age groups (11). A similar study examined platinum–taxane chemotherapy outcomes in the elderly, and with only half of elderly women getting platinum-based chemotherapy, an examination of treatment outcomes is warranted. The study found that age was not independently associated with outcomes in this 292-patient study of women with advanced ovarian cancer (50).

Finally, when examining treatment by physician type, elderly women seen by gynecologic oncologists were significantly more likely to receive adjuvant chemotherapy than those seen by general gynecologists and general surgeons (39).

### Recurrent Ovarian Cancer

In patients with advanced disease, nearly 85% will relapse even after adequate initial treatment (57). In these cases, treatment usually involves follow-up chemotherapy, avoiding surgery and surgery-related morbidities. To address this problem, a small study examined cytoreductive surgery and HIPEC in elderly women (57). No patients died immediately after surgery or from HIPEC-related complications. Median hospital stay was 13 days, with 20% of patients presenting G3–G4 complications. Median OS was 35 months, with median disease-free survival of 15.6 months. When the extent of carcinomatosis was assessed using the peritoneal cancer index (PCI), there were significant differences observed. For example, all patients with PCI >13 relapsed during the 2-year follow-up, and the authors concluded that in patients with PCI < 13, maximal cytoreductive surgery associated with HIPEC may improve the disease-free survival of elderly, recurrent ovarian cancer (57). Further studies with HIPEC are necessary, as it is a controversial treatment option with conflicting data.

The CALYPSO sub-study compared carboplatin–pegylated liposomal doxorubicin (C–PLD) with carboplatin–paclitaxel (C–P) in patients with late-relapsing recurrent ovarian cancer in elderly versus younger patients. While the elderly women had significantly fewer ≥Grade 2 allergic reactions, they had more ≥Grade 2 sensory neuropathy. Myelosuppression and completion rates of treatment did not differ between groups. Within the elderly patients, C–P was associated with more ≥Grade 2 alopecia, sensory neuropathy, arthralgia/myalgia, and severe leukopenia plus febrile neutropenia, while C–PLD was associated with more ≥Grade 2 hand–foot syndrome, providing a better therapeutic index with less toxicity in this elderly population (58).

The SOCRATES study assessed the pattern of care in patients with recurrent platinum-sensitive ovarian cancer at 37 Italian sites. Among the 493 patients analyzed, the recurrence-free interval (RFI), PS, and number of disease sites were similar between the elderly and younger women, but fewer elderly patients underwent secondary cytoreduction. The mean number of chemotherapy lines received for recurrence was similar, with the elderly patients more frequently receiving single-agent platinum at second line. The response rate to second-line chemotherapy was higher in younger patients, demonstrating a significant increase in median OS from recurrence. At multivariate analysis, age at recurrence was independently associated with survival, and the authors conclude that age is an unfavorable factor independently associated with a worse prognosis (59).

### Quality of Life

Quality of life (QoL) data available for review are extremely limited. The phase III AGO OVAR-3 trial evaluated QoL in elderly ovarian cancer patients using the European Organization for Research and Treatment of Cancer (EORTC) QoL questionnaire, and found no significant differences between the elderly and younger subgroups (14).

Also relating to QoL, in an analysis of over 8,000 elderly women with ovarian cancer, nearly 20% of women developed bowel obstruction after cancer diagnosis, of which all non-adhesion-related obstructions were considered pre-terminal events regardless of treatment type. Because of this, the authors suggest that patient comfort, not survival, should be the primary focus in this patient group (60).

## Conclusion

The data available for analysis regarding treatment outcomes in elderly ovarian cancer patients are conflicting; however, some general trends can be noted. As elderly women present more often with advanced stage (III–IV) disease, having prognostic tools to optimize treatment will be crucial in future care in this population. Most studies focused on the primary treatment for elderly women with ovarian cancer, with many suggesting that the aim should be focused on delivering optimal treatment, regardless of age. When providing suboptimal treatment to the elderly because of their age, numerous studies demonstrate suboptimal results with significantly lower survival outcomes. It would be important to develop tools to determine which elderly patients can actually tolerate aggressive therapy. While there is no consensus on whether age alone is an independent prognostic factor in this patient population, there seems to be consistency that optimal treatment (cytoreductive surgery with no residual disease remaining and combination chemotherapy) warrants further investigation in this population. To improve consistency among data, future studies should aim to determine an appropriate age defining “elderly.”

With a growing elderly population expected to double over the next couple of decades, further investigation into how to best treat this population is essential in optimizing future healthcare delivery to elderly women with ovarian cancer.

## Author Contributions

GF, ST, and DC all provided writing assistance and general support to SG in the preparation of the tables, figures, and drafting of the manuscript. All authors read and approved the final manuscript.

## Conflict of Interest Statement

The authors declare that the research was conducted in the absence of any commercial or financial relationships that could be construed as a potential conflict of interest.
